# Removing climbers more than doubles tree growth and biomass in degraded tropical forests

**DOI:** 10.1002/ece3.8758

**Published:** 2022-03-24

**Authors:** Catherine Finlayson, Anand Roopsind, Bronson W. Griscom, David P. Edwards, Robert P. Freckleton

**Affiliations:** ^1^ Ecology and Evolutionary Biology School of Biosciences University of Sheffield Sheffield UK; ^2^ 94205 Center for Natural Climate Solutions Conservation International Arlington Virginia USA

**Keywords:** biomass, climber, forest, liana, logged, tropic

## Abstract

Huge areas of tropical forests are degraded, reducing their biodiversity, carbon, and timber value. The recovery of these degraded forests can be significantly inhibited by climbing plants such as lianas. Removal of super‐abundant climbers thus represents a restoration action with huge potential for application across the tropics. While experimental studies largely report positive impacts of climber removal on tree growth and biomass accumulation, the efficacy of climber removal varies widely, with high uncertainty as to where and how to apply the technique. Using meta‐analytic techniques, we synthesize results from 26 studies to quantify the efficacy of climber removal for promoting tree growth and biomass accumulation. We find that climber removal increases tree growth by 156% and biomass accumulation by 209% compared to untreated forest, and that efficacy remains for at least 19 years. Extrapolating from these results, climber removal could sequester an additional 32 Gigatons of CO_2_ over 10 years, at low cost, across regrowth, and production forests. Our analysis also revealed that climber removal studies are concentrated in the Neotropics (*N* = 22), relative to Africa (*N* = 2) and Asia (*N* = 2), preventing our study from assessing the influence of region on removal efficacy. While we found some evidence that enhancement of tree growth and AGB accumulation varies across disturbance context and removal method, but not across climate, the number and geographical distribution of studies limits the strength of these conclusions. Climber removal could contribute significantly to reducing global carbon emissions and enhancing the timber and biomass stocks of degraded forests, ultimately protecting them from conversion. However, we urgently need to assess the efficacy of removal outside the Neotropics, and consider the potential negative consequences of climber removal under drought conditions and for biodiversity.

## Graphical abstract

Climber removal roughly doubles tree growth and triples aboveground biomasss accumulation in degraded tropical forests. The restoration method could provide substantial climate mitigation benefits.

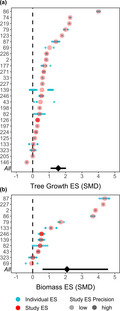

## 1 INTRODUCTION

Around 300 million hectares of moist tropical forest were deforested or degraded between 1990 and 2020 (Vancutsem et al., 2021). Both forms of disturbance threaten biodiversity, erode carbon stocks in a biome that contributes 55% of the global forest carbon sink, and reduce future timber yield, the main economic incentive for maintaining managed forests (Fisher et al., 2011; Gibson et al., 2011; Pan et al., 2011; Putz et al., 2012). While the protection of pristine ecosystems remains vital (Gibson et al., 2011), the enduring biological value of degraded forests emphasizes the critical role of restoration in conserving biodiversity, reducing atmospheric CO_2_, and supporting livelihoods (Edwards et al., 2014; Moomaw et al., 2019; Strassburg et al., 2020).

Various global initiatives, including the UN Decade on Ecosystem Restoration, the Bonn Challenge, and REDD+, recognize the benefits of restoration, with ambitions to restore hundreds of millions of hectares of degraded land (Cerullo & Edwards, 2019; Strassburg et al., 2020). However, “restoration” encompasses different strategies with varying potential, from converting agricultural land back to forest, to enhancing the state of degraded forests, such as those produced by selective logging (Moomaw et al., 2019; Strassburg et al., 2020). While restoring forests to currently nonforested land has huge potential (Strassburg et al., 2020), this is unlikely to yield the carbon sequestration required in the immediate future to meet global goals. Reforestation can also compete with food production and urban expansion (Moomaw et al., 2019). Alternatively, restoring degraded tropical forests to help them achieve their full ecological potential could remove approximately 350 PgCO_2_ from the atmosphere (Erb et al., 2018), recover timber stocks that prevents the expansion of “boom‐and‐bust” timber harvesting into pristine forests (Burivalova et al., 2020), and reduce the risk of degraded land being converted to more lucrative, but lower carbon and biodiversity value agricultural plantations (Cerullo & Edwards, 2019).

A key remaining question is how best to restore degraded forests (Coleman et al., 2019), and how much climate mitigation potential can be delivered, given large uncertainty in existing estimates (Griscom et al., 2017). A variety of methods have been developed for overall restoration of biodiversity and productivity in degraded forests, from “natural restoration” where human activity is simply removed, to enrichment planting where trees are planted to enhance natural restoration (Cerullo & Edwards, 2019). However, especially for enrichment planting, success and carbon gains can be limited, and interventions expensive (Burivalova et al., 2020; Philipson et al., 2020). An alternative solution is climber cutting. This method targets climbing plants such as lianas (woody, climbing plants) bamboo, and rattan that limit forest recovery. It is already widely recommended as part of reduced impact logging (RIL) practices, and is legally required but poorly implemented postlogging in Indonesia and other countries (Griscom et al., 2014; Putz et al., 2008; Ruslandi et al., 2017). Furthermore, climber cutting is relatively affordable (~$8.64 ha^−1^ across Africa and the Americas (see additional data) compared to enrichment planting (~$1500–$2500 ha^−1^ in Malaysian Borneo; Philipson et al., 2020)), requires limited expertise, can be easily integrated with forest inventories, and has potential to enhance forest restoration and carbon sequestration on a faster timescale (Cerullo & Edwards, 2019).

Climbing plants tend to proliferate extensively after disturbance and compete strongly with trees for light, water, and other resources, limiting tree growth, survival, recruitment, and aboveground biomass sequestration (Meunier, van der Heijden, et al., 2021; Meunier, Verbeeck, et al., 2021; Schnitzer & Bongers, 2002). Estrada‐Villegas and Schnitzer (2018) conclude that lianas have a negative impact on all metrics of tree performance, and it has been estimated that removing climbers in tropical forests enhances tree growth up to 372%, timber yield by 1.51 m^3^ per tree over 40 years, and aboveground biomass by ~76% per year compared to untreated forest (Estrada‐Villegas & Schnitzer, 2018; Mills et al., 2019; van der Heijden et al., 2015). However, these are site and region‐specific studies that report varying climber cutting efficacy.

Compared to untreated controls, the efficacy of climber cutting ranges from reducing tree growth by 20–90%, depending on size class (O’Brien et al., 2019), to more than doubling it (Gerwing, 2001; Grauel & Putz, 2004), with little consensus on what drives this variation. Marshall et al. (2017) noted that, across continents, tree growth after climber removal was enhanced by between 41% and 122% compared to control forest, but there is conflicting evidence regarding whether the outcome of climber removal on tree growth and carbon sequestration are influenced by region and climate. For example, two studies in SE Asia and Central America conclude that efficacy of cutting varies with total annual rainfall and between wet and dry seasons, while other studies find similar efficacy in wet and dry seasons (Álvarez‐Cansino et al., 2015; O’Brien et al., 2019; van der Heijden et al., 2019; Venegas‐Gonzalez et al., 2020).

Climber removal is also applied in various intensities and across different forest types, spanning old growth, selectively logged, and secondary forests of various ages, with no “best‐practice” procedures yet defined. In some cases, climber removal is applied just once to selected focal trees (Grogan & Landis, 2009), while in others removal is applied to the entire stand with repeated treatments (van der Heijden et al., 2019). Again, results are conflicting: some studies find a greater impact of climber removal on tree growth in younger forest, in earlier successional species, and on larger trees as climber load tends to be greater in these contexts (De Lombaerde et al., 2021; Duncan & Chapman, 2003; Estrada‐Villegas et al., 2020). Conversely, a recent study found no effect of liana removal on AGB accumulation across varying successional ages and tree sizes in a tropical dry forest (Estrada‐Villegas et al., 2021).

Due to the range in efficacy, breadth of climber removal contexts, and limited systematic attempt to understand drivers of variation in treatment efficacy, it is difficult to anticipate the outcome of climber removal with accuracy. Not only is this problematic for land managers, but it also limits our ability to estimate the contribution that climber removal could have to global restoration and carbon sequestration goals.

In this study, we use meta‐analytic techniques to determine the overall magnitude of climber removal efficacy in tropical forests, and to understand the potential drivers of variation in efficacy. We focus on tree growth and AGB accumulation as they contribute substantially to forest commercial value and productivity. We first synthesize existing experimental climber removal studies to quantify the effect of climber removal on enhancing tree growth and AGB accumulation, taking study context into account (Objective 1). We use this to estimate the potential contribution of climber removal to global carbon sequestration through restoration of degraded forests. Second, we exploit the breadth of study contexts to investigate whether region, climate, and forest disturbance context influence the efficacy of removal, to determine the best method of application, and to assess the longevity of treatment efficacy (Objective 2). Overall, this study determines whether climber removal can be applied to enhance aboveground biomass and timber stocks globally and, ultimately, restore function and economic value to degraded tropical forests.

## 2 METHODS AND MATERIALS

### 2.1 Literature search and screening

We conducted literature searches in Web of Science (WoS), SCOPUS, and Google Scholar, the latest search completed in March 2021. Author C.F. ran two search strings in each database: to find all studies that applied climber removal in tropical forests with any type of disturbance (none, regrowth after deforestation, and selectively logged), and to find studies that applied climber removal before disturbance (Appendix A: Table 1). We also conducted searches in the E‐Theses online Service (EThOS) database, contacted academics known to work on climber removal, and contacted organizations including national forestry departments and the Centre for International Forestry Research (CIFOR). This yielded a further 8 studies. Due to the high number of irrelevant results returned by Google Scholar, we screened results for relevance against inclusion criteria set a priori (Appendix A: Table 2) directly from the webpage. We stopped searching Google Scholar when we reached 100 consecutive irrelevant results. All WoS and SCOPUS search results were screened.

**FIGURE 1:**
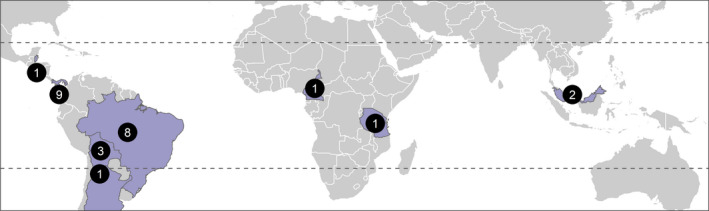
Geographical distribution of the 26 studies across the tropics included in the meta‐analysis literature search. A subset of these is included in the biomass analysis. Black circles indicate number of studies in each country. Dashed horizontal lines indicate the Tropic of Cancer (23° N) and the Tropic of Capricorn at (23° S)

The WoS, SCOPUS, relevant Google Scholar results, and the eight studies from other sources, yielded 5304 unique results. These were screened against the inclusion criteria, resulting in 65 studies (Appendix A: Figure 1). We then excluded 13 results that combined climber removal with another vegetation management, seven results that reused data from another publication, and six results that did not have a relevant tree growth or biomass metric (Appendix A: Table 3). A further 13 were excluded because mean tree growth, aboveground biomass (AGB), or control data were unavailable; authors were contacted for missing data before being excluded from the dataset. This resulted in 26 controlled experimental studies that assess the impact of climber removal on tree growth (Appendix A: Figure 1 and Table 4). For the AGB analysis, we only included a subset of the 26 studies which measured the effect of climber removal on trees ≥5 cm dbh, resulting in 12 studies. To quantify removal efficacy, we require treatment and control results for each study, contrasting to Estrada‐Villegas and Schnitzer (2018) that qualitatively summarizes 64 studies including noncontrolled studies and other responses to climber removal, such as tree mortality and canopy openness.

### 2.2 Data extraction

Author C.F. recorded data to calculate effect size (mean tree growth or AGB accumulation across all trees measured in treatment and control plots, variation around the mean, sample size [number of treatment and control plots], and tree growth response metric), study details (e.g., sampling effort and experimental design), and explanatory variables relating to region and climate, forest disturbance context, and method of removal that could influence climber removal. C.F. verified data at the time of extraction for accuracy. See Appendix B for details of tree growth and AGB response data collection, and details of how missing data were handled, and Appendix A: Table 4 and our published additional data for metadata of each study included in the analyses.

### 2.3 Meta‐analysis

#### 2.3.1 Calculating individual effect sizes

We calculated the individual effect sizes (ES) (and variance) for each study using the standardized mean difference (SMD; Hedges *g*) in relative growth rate (RGR) or AGB between treatment and control sites using the *metafor* and *compute*.*es* R packages (Del Re, 2013; Viechtbauer, 2010). Multiple effect sizes were calculated per study if there were treatment versus control comparisons measured at more than one timepoint, or on different size classes of trees. SMD is less biased by small sample sizes than mean difference (MD) and there was no difference in the results using either method (Appendix D: Figure 5). See Del Re (2015) for equations to calculate SMD and variance.

A value of SMD greater than zero indicates greater growth or biomass accumulation in trees in plots that had climbers removed compared to trees in control plots: the larger the positive number the greater the impact of climber removal. A value of SMD not significantly different from zero indicates equal tree growth or biomass accumulation in treated and control plots, meaning that climber removal has no significant effect.

#### 2.3.2 Assessing the magnitude of climber removal efficacy

To assess the magnitude of the effect of climber removal on promoting growth or biomass accumulation of trees (Objective 1), we fitted mixed‐effects linear models (using *lme4* and *lmerTest* R packages: Bates et al., 2015; Kuznetsova et al., 2017). One model was fitted to the 103 individual effect sizes from the 26 studies in the analysis of tree growth, and another to the 69 individual effect sizes from 12 studies in the analysis of biomass (Appendix C: Table 1). The models were run on each of the 10 datasets generated from imputing missing variances for growth and biomass (see Appendix B “Missing data” for details). The model results presented in the manuscript are the average parameter coefficients (including intercept), standard error of the coefficient, degrees of freedom, coefficient confidence intervals, and p‐values (based on these averaged values) from the 10 models. The models were weighted by the inverse SMD variance.

A unique study identifier was included as a random effect in both models to account for non‐independence when there were multiple effect sizes from each study. Time of measurement after treatment, number of species measured in mean growth rate, and study quality were included as fixed effects to capture known sources of variation between effect sizes or studies (Spake et al., 2020). Study quality is an ordinal scale (“high,” “medium,” or “low” quality), assigned based on study design, sample size, sampling effort (sampling area or number of trees measured), whether the tree growth was relative (RGR), how far the treatment site was from control plot, and whether there were any disturbance differences between treatment and control forests (Appendix B: Table 1). Study quality was included as a fixed effect as it only has three categories, and allows us to account for the variation between studies in terms of their design and rigor. The “number of species” variable accounts for variation caused by different studies measuring a different number of species, see Appendix B for more details.

We assessed the level of variation (heterogeneity) in the efficacy of climber removal using *Q* statistics and *I* ^2^ values. A significant *Q* statistic indicates significant heterogeneity, meaning that effect sizes from different studies vary more than would be expected by chance (Del Re, 2015; Harrison, 2011). The *I* ^2^ value indicates the extent of the heterogeneity, with 25% considered low, 50% considered moderate, and 75% considered a high amount of heterogeneity (Del Re, 2015).

#### 2.3.3 Assessing drivers of variation in climber removal efficacy

To determine whether region and climate, forest disturbance, or removal method were causing variation in climber removal efficacy (Objective 2), we added explanatory variables to the two models described previously. For the tree growth analysis, we included variables with the greatest theoretical impact on the outcome of climber removal (Appendix C: Table 1). The direction and size of the coefficient for each variable indicated its influence on climber removal efficacy. Several parameters could not be assessed (Appendix B: Table 2), or were assessed in supplementary models (Appendix C: Table 2), due to data constraints.

For the analysis of AGB, we were only able to assess the influence of a few parameters relating to removal method and disturbance context due to data constraints, and used three separate models to do so. We present all three models in the main text (see details in Appendix C: Table 1). Objective 2 models for tree growth and biomass accumulation were run for all imputed datasets (see Appendix B “Missing data” for details), and model results herein show the average parameter coefficients, standard error of the coefficient, degrees of freedom, coefficient confidence intervals, and *p*‐values (based on these averaged values). We assessed the heterogeneity of the Objective 2 models using *Q* and *I* ^2^ statistics.

### 2.4 Sensitivity analysis and assessing publication bias

We tested for publication bias in several ways. First, we analyzed the relationship between publication year and effect size to infer whether datasets with results opposing that of the first published paper remain unpublished. Second, we tested for asymmetry in funnel plots with Eggers test, using the *metafor* R package (Viechtbauer, 2010).

To test the robustness of the results, we calculated fail‐safe numbers following the Rosenthal, Rosenberg, and Orwin methods, using the *metafor* R package (Viechtbauer, 2010). These indicate how many studies with null results would need to be added to the analysis to reduce the significance level of the summary effect size so that it was no longer significant, or to reduce the effect size by half. Larger numbers indicate the effect size is robust.

### 2.5 Global carbon sequestration potential

To determine the potential contribution of climber removal to global carbon sequestration, we extrapolated the effect of climber removal on AGB accumulation (intercept of model for Objective 1.2) to an assumed maximum scenario. This includes: (a) the area of natural tropical forest managed for selective timber harvest with a valid concession license (282.9 million ha; FAO, 2020), and (b) the area of moist tropical forest regrowing >3 years after deforestation (29.5 million ha; Vancutsem et al., 2021). We calculated the difference between the baseline AGB growth rate for these forest types and the climber removal enhanced AGB growth rate. We then subtracted the AGB lost in removed climbing plants and their annual biomass growth, and converted the final difference in AGB to tons of CO_2_ (IPCC, 2003). See Table 3 and additional published data for full details.

All analyses were conducted in R (R Core Team, 2020) and figures produced using the R package *ggplot* (Wickham, 2016).

## 3 RESULTS

### 3.1 Global distribution and details of study sites

The 26 studies included in the analysis of tree growth are distributed across eight countries in the tropics, plus one in subtropical Argentina (−26 degrees latitude) (Figure 1). While there is good representation in Central and South America (22 studies), there were limited studies from Asia (2) and Africa (2). The 12 studies in the biomass analysis are from five countries, mainly in Central and South America (11 studies), plus Asia (1), with none in Africa.

The studies cover a range of elevations (range: 13–776 m.a.s.l), and gradients of precipitation (1144–2964 mm year^−1^), temperature (21.2–27.7°C), and dry season length (0–7 months). There were three studies in sites without any disturbance, 13 had been selectively logged, seven were forests regrowing after being cleared (secondary forest), and three were forests regrowing after being cleared that had also been selectively logged. Cutting was applied 1–720 months after disturbance in studies removing climbers post disturbance, and 1–12 months before removal for studies applying climber removal pre disturbance. Study monitoring duration ranged from 12 to 228 months post treatment. Studies repeated climber removal between 0 and 27 times, and applied removal across entire plots or just on focal trees. See Appendix A: Table 4 and additional published data for full study metadata.

### 3.2 Effect of climber removal on tree growth

**FIGURE 2:**
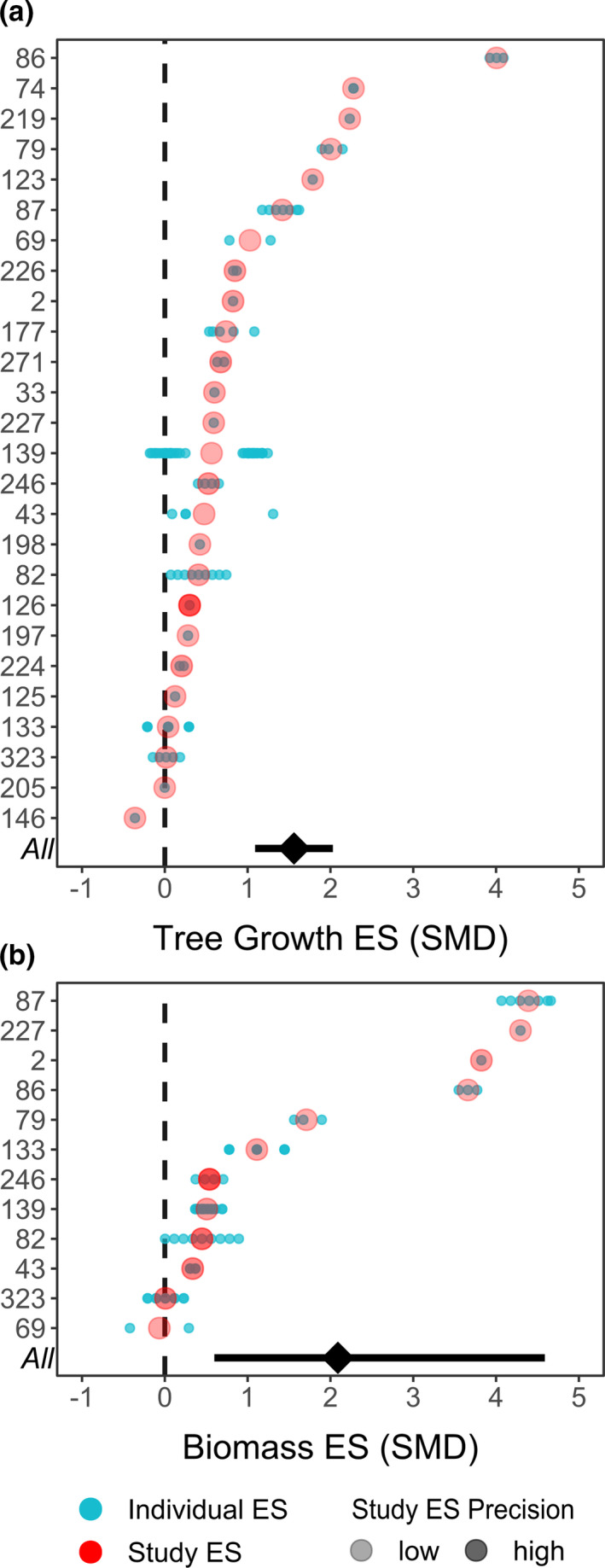
Overall, individual, and study average effect sizes (ES) of climber removal for promoting tree growth (Panel A) and AGB accumulation (Panel B). Numbers on the y‐axis represent study ID, as given in Appendix A: Table 4, and metadata spreadsheet in our published additional data. Blue dots are individual effect sizes within a study, predicted from the models for Objective 1.1 and 1.2 and averaged for all imputed datasets. Red circles are the study ES (the average of the individual ES for each study); the shade of the circle represents precision of the study ES and is proportional to the inverse of the variance of the individual effect sizes, averaged by study. The black diamond at the bottom of each figure is the overall summary effect size of climber removal for promoting tree growth and biomass, taken from the intercept of the models for Objective 1.1 and 1.2 when continuous covariates are at their mean value and study quality reference level is “high”; error bar shows 95% credible intervals

**FIGURE 3:**
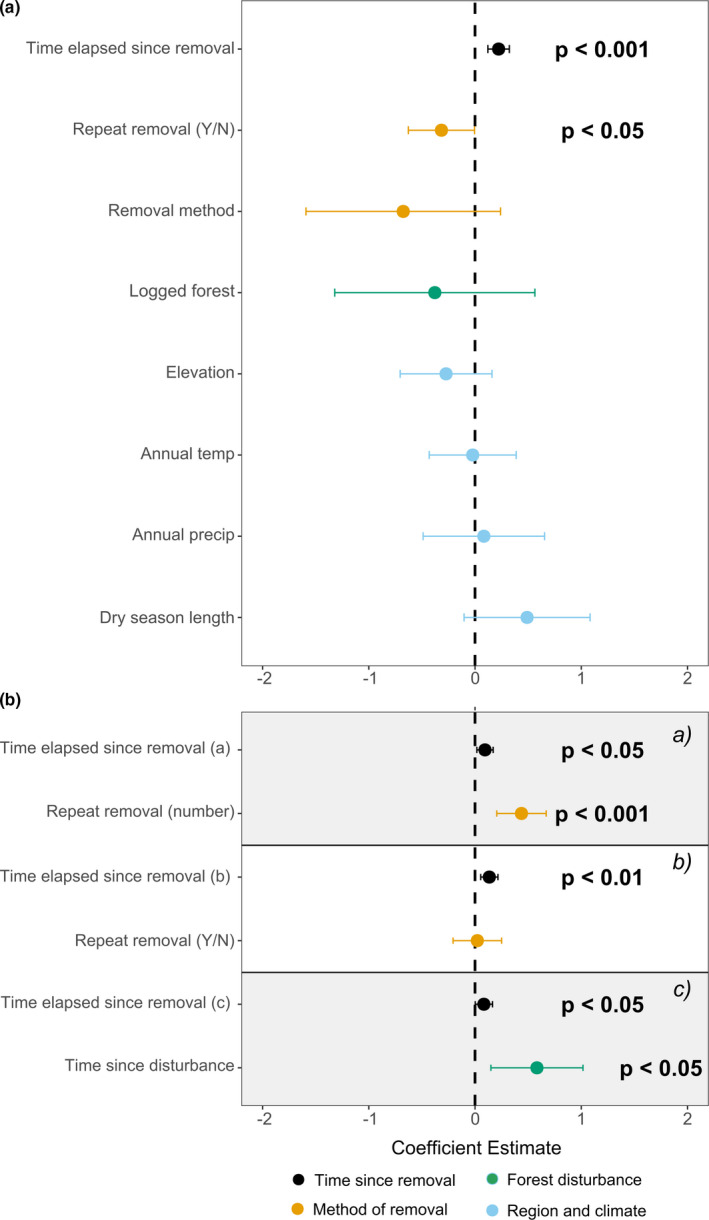
Influence of region and climate, disturbance context, and method of removal (whole plot vs focal tree removal and whether removal was repeated) on the efficacy of climber removal for promoting tree growth and AGB accumulation. Panel A shows coefficient estimates for the Objective 2.1 (tree growth) model and Panel B shows estimates for the Objective 2.2 (AGB) models a, b, and c. The coefficient for the repeat removal (number) in model 2.2 c) is not shown in the figure as it was no different from model a). Centred and scaled parameter estimates are shown for continuous variables with error bars indicating 95% CI. For categorical variables, the figure shows the fitted mean value with 95% CI between the reference level and the other categorical level. The reference level for the “Logged forest” variable is “logged,” “Repeat removal (Y/N)” variable is “no repeated removal,” and “Removal method” variable is the whole plot removal method. Significant parameter estimates are shown with *p*‐values. Color indicates the parameter category

We find that the results of our meta‐analysis are robust, even though there is some evidence of publication bias (see Appendix D: Figures 1–4 and text for details). Trees in plots from which climbers were removed experienced a 2.56‐fold increase in growth (summary effect size 156%; 95% CI = 109–203%) compared to those in untreated control plots (Figure 2, Table 1) across all tree size classes and various growth metrics combined. This represents the tree growth enhancement resulting from climber removal at the stand level. There was substantial variation in the effect on tree growth: the lowest individual effect size across studies showed a −36% decrease in tree growth, whereas the highest showed a 409% increase in growth. African studies had effect sizes of −36% and 12%, and Asian studies had effect sizes of 56% and 179% compared to untreated controls (Lussetti et al., 2016; Marshall et al., 2017; O’Brien et al., 2019; Parren, 2003, respectively; Figure 2). The median effect size outside the Neotropics (29%) is much lower than the overall tree growth effect size (156%), but we could not directly assess the influence of region due to insufficient studies located in Asia and Africa (see *Methods* section 3.3).

**TABLE 1:** Magnitude and direction of climber removal efficacy on tree growth and biomass accumulation

Objective	Fixed effect	Estimate (SE)	Degrees of Freedom
Objective 1.1: Tree growth	Tree growth ES	1.56 (0.23)***	32
	Study quality High:Low	−1.22 (0.40)**	81
	Study quality High:Med	−1.18 (0.15)***	86
	Number of species	0.00 (0.00)	89
	Time elapsed since removal	0.01 (0.00)***	90
Objective 1.2: AGB accumulation	AGB ES	2.09 (0.67)*	11
	Study quality High:Low	−1.97 (1.76)	7
	Study quality High:Med	−0.23 (0.41)	61
	Number of species	−0.00 (0.01)	8
	Time elapsed since removal	0.01 (0.00) *	54

Note

Results of models for Objective 1.1 (tree growth) and Objective 1.2 (AGB). Estimates for “Tree growth ES” and “AGB ES” are the intercept of each model and show the increase in tree growth or biomass accumulation in climber removal versus untreated control plots. Results are the average of 10 Linear Mixed Models using 10 datasets imputed using linear regression, including the study with just post‐treatment data (Tree growth *N* = 26 studies, Biomass *N* = 12 studies). See Appendix C for full description of models. Bolded estimates indicate level of significance at either 0.05, 0.01, or 0.001.

*p < .05, **p < .01, ***p < .001.

*Q* statistics and *I* ^2^ values indicate that the magnitude of the positive effect of climber removal on enhancing tree growth is expected to vary, but only by a small amount (*Q* = 164, 95 CI = [121–218], *p*‐val < .001; *I* ^2^= 38%, 95% CI = [16–53%]). Model results do not differ substantially if we excluded imputed data, if we calculated effect sizes using MD rather than SMD (Appendix D: Figure 5), or if we removed van der Heijden et al. (2015) that had an effect size almost double those of the other studies (Appendix D: Table 1).

The efficacy of climber removal for enhancing tree growth varied with quality of study: efficacy was 122% greater (95% CI = [44, 201]) in high‐ than low‐quality studies, and 118% greater (95% CI = [88, 149%]) in high‐ than medium‐quality studies (Table 1). We observed that the efficacy of climber removal for enhancing tree growth did not vary with the number of species in the mean growth rate (Table 1).

### 3.3 Effect of climber removal on AGB accumulation

Climber removal increased total aboveground biomass storage of all trees in treated plots by 3.09 times (summary effect size 209%; 95% CI = [60, 359%]) compared to untreated controls. This represents the increased AGB accumulation resulting from climber removal at the stand level. Again, there was substantial variation, with the individual effect size sizes across studies ranging from −42 to 466% (Figure 2, Table 1). The only study outside the Neotropics (in Malaysia) experienced 51% increase in AGB compared to untreated controls. The effect size was much lower and the credible intervals cross zero when imputed data is not included (*N* = 9) (Appendix D: Figure 6), but only one study of nine had a negative effect of climber removal on biomass, confirming the overall positive effect of climber removal on biomass accumulation. *Q* statistics and *I* ^2^ values indicate that, while we expect a positive effect of climber removal, the magnitude of the effect of climber removal on AGB accumulation is likely to vary substantially (*Q* = 257, 95 CI = [150, 371], *p*‐val < .001; (*I* ^2^= 74%, 95% CI = [55, 82%]).

### 3.4 Drivers of variation in efficacy for tree growth

Explanatory variables relating to climate, region, and forest disturbance did not influence the efficacy of climber removal for enhancing the growth of trees (Figure 3, Table 2). However, efficacy did increase, marginally, per month since treatment (1% greater effect on tree growth per month in Objective 1.1 and 2.1 models (95% CI = [0, 1%]); Tables 1 and 2). This shows that climber removal enhances tree growth for at least the maximum study monitoring period of studies in this analysis: 19 years. The model for Objective 2.1 found that studies which repeated removal had 41% less tree growth enhancement compared to studies which did not repeat removal (95% CI = [1, 82%]; Table 2). However, the confidence intervals are very close to zero and the supplementary models suggest that repeating removal does not significantly influence the efficacy of climber removal for enhancing tree growth (Appendix D: Table 2). Supplementary models also found no effect of latitude, time between disturbance and removal, and dry season temperature and precipitation on the efficacy of climber removal for promoting tree growth.

**TABLE 2:** Drivers of variation in the efficacy of climber removal for tree growth and AGB accumulation

Objective		Explanatory parameter	Estimate (SE)	Degrees of Freedom
Objective 2.1 (Tree growth)		Time elapsed since removal	0.01 (0.00)***	86
		Repeat removal (Y/N)	−0.41 (0.20)*	91
		Removal method (whole plot/focal tree)	−0.88 (0.57)	21
		Logged forest	−0.49 (0.58)	17
		Dry season length	0.30 (0.17)	17
		Annual precipitation	0.00 (0.00)	16
		Annual temperature	−0.02 (0.19)	19
		Elevation	−0.00 (0.00)	23
Objective 2.2 (AGB accumulation)	(a)	Time elapsed since removal	0.01 (0.00)*	54
		Repeat removal (number)	0.18 (0.05)***	62
	(b)	Time elapsed since removal	0.01 (0.00)**	54
		Repeat removal (Y/N)	0.04 (0.27)	56
	(c)	Time elapsed since removal	0.01 (0.00)*	48
		Repeat removal (number)^a^	0.17 (0.05)***	51
		Time since disturbance	1.16 (0.40)*	13

Note

Results for Objective 2.1 and 2.2 models, averaged from 10 Linear Mixed Models using 10 imputed datasets (imputed using linear regression), and including one study with just post‐treatment data (tree growth *N* = 26 studies, biomass *N* = 12). Response variable is tree growth for Objective 2.1 and AGB change for Objective 2.2, see full model details in Appendix C. Bolded explanatory parameters indicate level of significance at either .05, .01, or .001. *p < .05, **p < .01. ***p < .001.

^a^ Excluded from Figure 3 as the same result as model a.

As with Objective 1.1, the *Q* statistics and *I* ^2^ values indicate that the positive effect of climber removal on tree growth is still likely to vary by a small amount, even when accounting for variation due to parameters included in the model for Objective 2.1 (*Q* = 177, 95 CI = [132, 232], all *p*‐values < .001; *I* ^2^ 42%, 95% CI = [23, 56%]).

### 3.5 Drivers of variation in efficacy for AGB accumulation

The AGB accumulated in treated plots relative to untreated plots increased with the time elapsed since removal, the number of times the treatment was applied, and the amount of time between disturbance and initial application of removal (Tables 1 and 2, Figure 3). The efficacy of climber removal for enhancing AGB increased 0.1% (95% CI = [0.0, 1.2%]) with each month elapsed since removal. This shows that climber removal enhances AGB for at least 10 years: the maximum study monitoring period of studies in the biomass analysis. We also found that removal more greatly enhanced biomass accumulation in older secondary forest and forests logged longer ago: efficacy increased by 115.9% (95% CI = [29.7, 202.0%]) with each additional year between disturbance and treatment (maximum 60 years between disturbance and treatment). Efficacy also increased by 18% with each removal repetition (95% CI = [9, 28%]).

According to the *Q* statistics and *I* ^2^ values, the positive effect of climber removal on AGB accumulation is still expected to vary substantially, even when accounting for variation due to parameters included in the models for Objective 2.2 (Q = 239–269, 95 CI = [132–383], *p*‐val < .001; *I* ^2^ = 65–68%, 95% CI = [41, 84%]; across Objective 2.2 a, b, and c models).

### 3.6 Global carbon sequestration potential

Extrapolating the 209% increase in AGB accumulation resulting from climber removal to our assumed maximum application scenario (timber production and secondary forest), we find that climber removal could sequester an additional 32 Gigatons of CO_2_ over a decade (22.9 in production forest and 9.2 in secondary forest; Table 3). With the mean reported cost of climber removal as US$8.64 ha^−1^ (see additional published data), we calculate the cost of climber removal as US$0.11 and US$0.03 per Mg (metric ton) of CO_2_ sequestered over 10 years for selectively logged and secondary forests, respectively (range: US$0.003–US$0.22; Table 3).

**TABLE 3:** Global carbon sequestration potential of climber removal

Forest Classification	AGBg_0_ (Mg C ha^−1^ yr^−1^)^a^	AGBg_CR_ (Mg C ha^−1^ yr^−1^)^b^	Area of forest (ha)^c^	Additional carbon sequestration with climber removal over 10 years (Mg C0_2_)^d^	Cost of climber removal per CO_2_ sequestered over a decade (US$ Mg CO_2_^−1^) (min,max)^e^
Production forests	1.49	4.61	282,879,090	22,862,805,785.40	0.11 (0.01,0.22)
Secondary Forests >3 years since deforestation	4.49	13.87	29,500,000	9,163,581,171.56	0.03 (0.003,0.06)
Total			312,379,090.00	32,026,386,956.95	

Note

Extrapolating the enhancement of AGB accumulation through climber removal (intercept of the model for Objective 1.2) to calculate the carbon sequestration that could be provided by climber removal in production and secondary tropical forests. See published additional data for full calculation.

^a^ AGB*g_0_* is the baseline biomass growth (in metric tons [Mg] of carbon per hectare per year): for production forest this is the mean biomass growth rate from Butarbutar et al. (2019), Gourlet‐Fleury et al. (2013), and Rutishauser et al. (2015); for secondary forest this is the mean from Cook‐Patton et al. (2020).

^b^ *AGBg_CR_* is the climber removal enhanced biomass growth rate: $\text{AGB}g_{0}+\left(\text{AGB}g_{0}*2.09\right)$ [effect of climber removal on AGB accumulation; intercept of model for Objective 1.2].

^c^ Forest area classified as production forests with a valid concession license (designated management Objective or production forests; FAO, 2020); Area of moist tropical forest that is classified as regrowing >3 years post deforestation event (Vancutsem et al., 2021).

^d^ Difference in baseline and enhanced AGB growth for 10 years in each area of forest, accounting for biomass lost from removing climbers (AGB_climber_) and converted to CO_2_ as per IPCC, 2003 guidelines: $\left(\left(\text{AGB}g_{\text{CR}}*\text{Area}*10\right)‐\left(\text{AGB}g_{0}*\text{Area}*10\right)‐\left({\text{AGB}}_{\text{climber}}*\text{Area}*10\right)\right)*44/12$.

^e^ Cost of climber removal per each additional metric ton of CO_2_ sequestered in a decade: See additional published data for total cost of climber removal in production and secondary forests.

## 4 DISCUSSION

Quantifying the benefits of climber removal for tree growth and AGB is crucial for deciding whether removal should be implemented. We find that climber removal more than doubles tree growth and roughly triples AGB accumulation compared to untreated forests, and that efficacy is sustained for at least 19 years. We also quantify the potential of implementing climber removal for global carbon sequestration and provide recommendations for applying climber removal in certain regions, but note the lack of evidence outside the Neotropics and highlight urgent areas for research.

### 4.1 Climber removal substantially enhances tree growth and AGB accumulation

Our results confirm the findings of individual studies that climber removal has an overall positive effect on tree growth and AGB accumulation (Estrada‐Villegas & Schnitzer, 2018), plus emphasize the dramatic role of climbers in tropical forest growth dynamics, carbon sequestration, and forest management. Our approach builds on the largely qualitative Estrada‐Villegas and Schnitzer (2018) review by calculating the size of the effect of climber removal and uncertainty in efficacy, while methodically accounting for study context. We also estimate the potential contribution of climber removal to global carbon sequestration: sequestering 32 Gigatons of CO_2_ in a decade at relatively low cost if applied to secondary forests and production forests across the tropics.

The global carbon sequestration potential is not surprising given the unrealized carbon potential of degraded tropical forests (350 Gigatons CO_2_) identified by Erb et al. (2017). However, our extrapolation may be influenced by (i) selection bias for studies occurring in locations with high climber density, (ii) our inclusion of a few studies that only measure the efficacy of removal on trees directly infested with climbing plants (rather than all trees in a given plot), and (iii) extrapolating to the total area of secondary forests >3 years old while our analysis only included studies conducted in secondary forests 20–60 years old. On the other hand, climbers do influence entire plots, not just the tree they infest (van der Heijden et al., 2015), and climber infestation in degraded forests tends to be high (Schnitzer & Bongers, 2002; up to 80% trees infested in selectively logged forest in Malaysian Borneo [*unpublished data*]). Moreover, secondary forests only contribute a third of the total calculated sequestration potential of climber removal, and we do not account for the reduced tree mortality and enhanced seedling recruitment associated with climber removal (Philipson et al., 2020; van der Heijden et al., 2015; but see O'Brien et al., 2019). For these reasons, we anticipate that any over‐estimate of the climate mitigation potential of climber removal is limited. Nevertheless, more research and more detailed data, such as climber abundance and individual tree‐level data, are needed to further refine our global estimates of the stand‐level impact of climber removal on tree carbon sequestration rates.

This study demonstrates how to extrapolate our results to the extent of tropical forest in two scenarios. Our estimate assumes that the maximum extent where climber removal is appropriate is 312.4 million ha (tropical timber production natural forest and secondary forest). While it will not be feasible in every hectare in these landscapes, and many logging concessions are not yet logged nor will see the benefit of climber removal for some time, we consider this a conservative estimate. We restrict our tropical timber production forest to areas under valid timber concession licenses (282.9 million ha), while noting there is a larger area reported as production forest (~400 million ha according to FAO (2020)). Further, Potapov et al. (2017) estimate ~1.4 billion ha is non‐intact tropical forest, indicating considerably larger maximum extent for implementing climber removal. Using our study as an example, extrapolations could be made for alternative forest extents, at scales relevant to individual countries or landowners, and regarding timber rather than carbon stocks.

### 4.2 Influence of region and climate remains unclear

Though our results give no indication that the efficacy of climber removal on tree growth and biomass accumulation is influenced by elevation, latitude, presence, and length of dry season, precipitation, and temperature, the poor distribution of study sites means there is insufficient evidence to conclude that region and climate have no effect. There are very few studies outside the Neotropics, none in the montane tropics and forests with the highest annual rainfall (e.g., the Chocó, Colombia), and very few studies considered the influence of drought, despite their increasing frequency and concerns that climber removal may be detrimental in drought conditions (Berenguer et al., 2021; IPCC, 2021; O’Brien et al., 2019). The scarcity of climber removal studies outside the Neotropics represents a major gap in our knowledge: particularly troubling as climber removal is increasingly prescribed as a restoration intervention (Cerullo & Edwards, 2019; Philipson et al., 2020).

Climber removal, nonetheless, remains an important potential restoration action, especially in Africa and Asia where forest disturbance is widespread and climber abundance is high (DeWalt et al., 2014; Hansen et al., 2013). Removal studies in these regions and across wider climatic gradients are urgently required so that evidence‐based climber removal can be rolled out pan‐tropically. Beyond the tropics, and outside the scope of this meta‐analysis, climber removal could also be important in temperate regions, where competing vegetation and climber abundance can hinder growth and biomass accumulation (De Lombaerde et al., 2021; Smith, 1984).

### 4.3 Efficacy of climber removal is similar across disturbance history and methods of removal

Overall, we find limited evidence that the efficacy of removal is influenced by forest disturbance context or method of removal. Climber removal enhances tree growth to a similar extent in selectively logged and secondary forests disturbed up to 60 years previously. This confirms that climber competitive advantage is similar in both selectively logged and secondary forests, and sustained long after disturbance (Schnitzer & Bongers, 2002). Furthermore, our results suggest that sufficient climbers are removed to enhance tree growth with a single intervention and when limited to focal trees. The number of removal interventions and intensity of removal (focal tree or whole plot removal) are key considerations when applying climber removal (Gerwing, 2001; Grogan & Landis, 2009; Mills et al., 2019).

While our biomass analysis found that AGB accumulation was more enhanced by climber removal in forests disturbed longer ago and when removal is repeated, the strength of our conclusions is limited by the number of studies (*N* = 12). However, given that the abundance of larger trees increases with age of forest, and that only trees >5 cm dbh were included in the biomass analysis, this result could indicate that larger trees benefit more from climber removal, potentially due to higher climber loads (Estrada‐Villegas et al., 2021). Moreover, the four studies with higher biomass effect sizes in Figure 2 all experienced disturbance at least 55 years ago, or were undisturbed, highlighting the need to corroborate the influence of time since disturbance on removal efficacy.

### 4.4 Recommendations for application and conclusions

We identify two key climber removal scenarios for timber and carbon benefits in the Neotropics. First, in timber production forests, forestry personnel could apply removal to just focal trees, during preharvest inventory and timber cruising for greatest efficiency. This is especially significant considering the huge area of production forests (FAO, 2020). Second, a single application of “whole‐plot” climber removal could be conducted by unskilled labor in degraded forests (regrowing or already selectively logged). Edges of forests could be specifically targeted as they have low value and are easy to access (Ordway & Asner, 2020; Poor et al., 2019), though the important role of climbing plants in edge forests should not be jeopardized (Magnago et al., 2017). Moreover, prioritizing removal in older regrowth forests would yield the highest AGB accumulation rates as regrowing forests have higher baseline sequestration rates than selectively logged forests (Butarbutar et al., 2019; Cook‐Patton et al., 2020; Gourlet‐Fleury et al., 2013; Rutishauser et al., 2015).

The expected gains in growth rates in these scenarios will ultimately contribute to climate mitigation, enhance sustainable timber yields, potentially limit the expansion of timber harvesting into primary forest (Burivalova et al., 2020), and enhance the economic value and function of degraded forests that may prevent their conversion (Cerullo & Edwards, 2019). However, while preventing degraded forests from conversion could protect biodiversity, this study only considers the impact of climber removal on tree and AGB growth, ignoring the various functions of climbing plants in tropical forests. Their removal could have negative consequences for biodiversity, for example reducing the species richness of climbing plants, removing food and locomotion resources, and influencing the microclimate (Addo‐Fordjour et al., 2020; Arroyo‐Rodriguez et al., 2015; Campbell et al., 2015; Cosset & Edwards, 2017; Magnago et al., 2017; Putz et al., 2001; Schnitzer et al., 2020), though see Cerullo et al. (2019). Our study finds that applying removal just to focal trees and not repeating treatment yield growth benefits while giving climbers greater chance to recover, but this will not be enough to prevent biodiversity losses from climber removal. Additional best‐practice guidelines, such as leaving areas of forest untreated and avoiding certain climber species, are critical to safeguard the functional role of climbing plants and minimize negative impacts on biodiversity.

While it may not be feasible, nor advisable, to apply climber removal across the entire tropics, this action clearly presents a major climate mitigation opportunity: one that has not been accounted for in prior estimates of natural climate solutions (Griscom et al., 2017, 2020; Roe et al., 2021). We recommend that climber removal is implemented to some extent as part of restoration and carbon sequestration programs in the Neotropics, specifically as part of forest management in logging concessions, pre‐ and postharvest, and in already degraded forests. However, further studies are urgently required to confirm treatment efficacy in Africa and Asia, and to minimize negative biodiversity implications of climber removal. With climber removal, we have the potential to greatly improve the value of degraded tropical forests, and the future of global biodiversity and carbon sequestration.

## CONFLICT OF INTEREST

The corresponding author confirms on the behalf of all authors that there are no competing interests.

## AUTHOR CONTRIBUTIONS

**Catherine Finlayson:** Conceptualization (equal); Data curation (lead); Formal analysis (lead); Methodology (equal); Visualization (lead); Writing – original draft (lead). **Anand Roopsind:** Data curation (supporting); Methodology (supporting); Writing – review & editing (equal). **Bronson W. Griscom:** Data curation (supporting); Methodology (supporting); Writing – review & editing (equal). **David P. Edwards:** Conceptualization (equal); Data curation (supporting); Formal analysis (supporting); Funding acquisition (equal); Methodology (supporting); Supervision (equal); Visualization (supporting); Writing – review & editing (equal). **Rob P. Freckleton:** Conceptualization (equal); Data curation (supporting); Formal analysis (supporting); Funding acquisition (equal); Methodology (supporting); Supervision (equal); Visualization (supporting); Writing – review & editing (equal).

### OPEN RESEARCH BADGES

This article has earned an Open Data Badge for making publicly available the digitally‐shareable data necessary to reproduce the reported results. The data is available at https://doi.org/10.5061/dryad.zs7h44jb2.

## Supporting information

Appendix S1

## Data Availability

All data that are necessary to reproduce the results presented in this study, the metadata of studies included in the analyses, and details of the global carbon extrapolation are available on Data Dryad at https://doi.org/10.5061/dryad.zs7h44jb2.
