# Corrigendum

**DOI:** 10.1002/ece3.9917

**Published:** 2023-03-24

In the article by Finlayson et al. (2022), titled “Removing climbers more than doubles tree growth and biomass in degraded tropical forests”, the authors note an error. The paper misinterprets the standardized mean difference (SMD) summary effect size, resulting in an overestimation of the effect of climber removal on tree growth and biomass and an overestimation of the global carbon sequestration potential of climber removal. This correction finds that climber removal *almost doubles* aboveground biomass (AGB) accumulation in degraded tropical forests rather than tripling AGB.

Throughout the article, SMD should be interpreted as the number of standard deviations difference between the tree growth and AGB in treated and control forest plots rather than the proportional effect of climber removal. For example, the summary SMD for tree growth of 1.56 means tree growth is 1.56 standard deviations higher in treated than control plots, rather than a 2.56‐fold (or 256%) increase in tree growth, and the summary SMD for AGB of 2.09 means AGB is 2.09 standard deviations higher in treated than control plots, rather than a 3.09‐fold (or 309%) increase.

In this correction, we use the log response ratio (logRR) between climber removal treated and control plots rather than the SMD to calculate the *proportional* effect of climber removal on biomass accumulation and the global carbon sequestration potential of climber removal. We calculate a summary log response ratio (logRR) of 0.63, equating to an 88% increase in biomass accumulation (95% CI = 40%–145%) in climber removal relative to control plots once logRR is transformed back to a normal scale. Extrapolating this proportional effect to timber production and secondary forests across the tropics, we find that climber removal could sequester an additional 7.4 Gigatons of CO_2_ over a decade (4.1 in production forest and 3.3 in secondary forest) at a cost of US$0.59 and US$0.08 per Mg (metric ton) of CO_2_ sequestered over 10 years, respectively (range: US$0.01–US$1.19).

The overall conclusion of the paper remains the same: There is a significant and substantial positive effect of climber removal on tree growth and aboveground biomass compared with untreated forest stands.
