# Supplementary material for: Removing climbers more than doubles tree growth and biomass in degraded tropical forests
Source: Ecol Evol. 2022 Mar 24;12(3):e8758. doi: 10.1002/ece3.8758 (PMC8948070; doi:10.1002/ece3.8758)
Supplement: Supplementary file 1 — Appendix S1 [file ECE3-12-e8758-s001.docx]

# Appendix A: Literature search and screening

**Table 1: Literature search strings for Web of Science, SCOPUS and Google Scholar.** Two different search strings per database.

| **Pre disturbance/ any** | **Database** | **Search string** | **Further refinement ^a^** |
| --- | --- | --- | --- |
| Any | Web of Science (All databases) | TOPIC: ((liana* OR vine* OR climb*) AND (remov* OR cut* OR clear* OR thin* OR liberat* OR experiment*) AND (forest*), NOT vineyard, NOT medical | - Refined by Science and Technology - Excluding patent and clinical trials and engineering research domain |
| Any | SCOPUS | ( TITLE-ABS-KEY ( liana* OR vine* OR climb* ) AND TITLE-ABS-KEY ( remov* OR cut* OR clear* OR thin* OR liberat* OR experiment* ) AND TITLE-ABS-KEY ( forest* ) AND NOT TITLE-ABS-KEY ( vineyard ) AND NOT TITLE-ABS-KEY ( medical ) ) AND ( EXCLUDE ( SUBJAREA , "ENGI" ) ) | - Exclude Social Sciences and Engineering |
| Any | Google Scholar | (liana OR vine OR climb) AND (remove OR cut OR clear OR thin OR liberat OR experiment) AND (forest) | - |
| Pre | Web of Science (All databases) | TOPIC: ((“pre-disturb*” OR “pre-log*” OR “pre-fell*” OR “pre-harvest*” OR “pre-exploit*” OR prefell* OR “prior to log*” OR “prior to disturb*” OR “prior to fell*” OR “prior to harvest*” OR “prior to exploit*”)  AND ((liana* OR vine* OR climb*) AND (remov* OR cut* OR clear* OR thin* OR liberat* OR experiment*)) OR “thinning operation*”)  OR TOPIC: (RIL OR "reduced-impact log*" OR “silvicultur* field experiment” OR “planned fell*” OR “planned log*” OR “planned harvest*” OR “FSC cert*”)  OR TOPIC: (“silvicultur* treatment*” AND (liana* or vine* OR climb* OR RIL OR "reduced-impact log*")) | - Refine by (forest* OR concession* OR “logging operation”) and Science/Technology - Exclude Patents/News |
| Pre | SCOPUS | ( TITLE-ABS-KEY ( ( ( "pre-disturb*" OR "pre-log*" OR "pre-fell*" OR "pre-harvest*" OR "pre-exploit*" OR prefell* OR "prior to log*" OR "prior to disturb*" OR "prior to fell*" OR "prior to harvest*" OR "prior to exploit*" ) AND ( ( liana* OR vine* OR climb* ) AND ( remov* OR cut* OR clear* OR thin* OR liberat* OR experiment* ) ) OR "thinning operation*" ) OR ( ril OR "reduced-impact log*" OR "silvicultur* field experiment" OR "planned fell*" OR "planned log*" OR "planned harvest*" OR "FSC cert*" ) OR ( "silvicultur* treatment*" AND ( liana* OR vine* OR climb* OR ril OR "reduced-impact log*" ) ) ) ) AND ( forest* OR concession* OR "logging operation" ) | - Exclude Social Sciences |
| Pre | Google Scholar | (("pre-logging" OR "pre-harvest" OR "prior to logging") AND (liana OR vine) AND (removal OR cut OR cutting OR liberation) OR "thinning operation") OR RIL OR "reduced impact logging" OR "planned logging" OR "FSC certified" OR "silviculture treatment" | - - |
| ^a^ Any further filtering applied to the search results after using the indicated search string. | | | |

**Table 2: Inclusion criteria.**

| **PICO^a^** | **Inclusion criteria** |
| --- | --- |
| Population | Tropical (latitudes between 26°C North and South, inclusive)  Selectively logged (i.e., never fully clear-cut), secondary (clear-cut and regrowth) and undisturbed forest  Natural forest system, i.e., not the following:   - - Tree plantation   - Vineyard   Not mangrove forest  Experimental removal of climbers; not modelling paper |
| Intervention | Climber removal (by any method such as cutting with machete or poisoning) explicitly applied |
| Comparator | Control sites in tropical forest with the same level of disturbance in which no climber removal was applied |
| Outcome | Tree growth (diameter, basal area, biomass, height, etc.) |
| ^a^ PICO elements are used to structure a search strategy and eligibility criteria aimed at answering a specific question (Livoreil *et al.*, 2017). | |


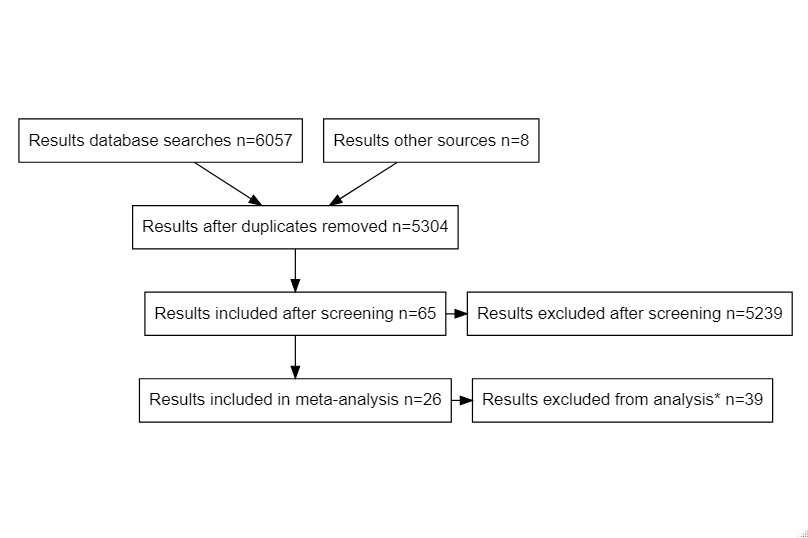


**Figure 1:** Flow chart illustrating literature search and screening process.

*These 39 results were excluded for the following reasons: additional vegetation management, duplicated data, not useful tree growth metric, mean growth data unavailable for climber removal and/or control treatments.

**Table 3: Studies not included in meta-analysis after data extraction and full text assessment.**

| **Author and year** | **Excluded reason category** | **Reason, full** |
| --- | --- | --- |
| Cardoso et al 2014 | Additional vegetation removal | Additional silvicultural treatment other than just climber or understory removal (e.g., thinning of competing trees or planting of seedlings) |
| Heuberger et al 2002 |  |  |
| Pena-Claros et al 2002 |  |  |
| Putz et al 1984 |  |  |
| Schiotz et al 2006 |  |  |
| Schwartz et al 2013 |  |  |
| Villegas et al 2009 |  |  |
| Guaragiata 1999 |  |  |
| Butarbutar et al 2019 |  |  |
| Coimbra Cordeiro et al 2019 |  |  |
| Oliveria et al (unpublished thesis chapter) |  |  |
| Minh Quang et al 2020 |  |  |
| Truong et al 2021 |  |  |
| Schnitzer et al 2014 | Duplicate data | Only data available (biomass growth) represents the same tree growth (using the same plots and treatments) as Schnitzer et al, 2010 |
| Schnitzer et al 2004 |  | Duplicate data using same plots and treatments as Parren, 2003 |
| Taffarel and de Carvalho et al 2014 |  | Only has growth data for individual species that contribute to overall growth in de Souza et al, 2015 |
| Vatraz et al 2012 |  | Only has growth data for individual species that contribute to overall growth in de Souza et al, 2015 |
| Venturoli and Carvalho et al 2015 |  | Duplicate data from same experimental sites in Freitas Xavier et al, 2017 |
| Venturoli and Franco et al 2015 |  | Duplicate data from same experimental sites in Freitas Xavier et al, 2017 |
| Estrada-Villegas 2019 |  | Duplicate data in Estrada-Villegas et al, 2020 |
| Douglas 1996 | Not useful growth metric | Only has data for post-treatment size of tallest tree |
| Campanello et al 2012 | Not useful growth metric: Net growth | Tree growth in basal area of entire plot, could include recruitment |
| Forshed 2006 |  | Net growth including recruitment and mortality of trees |
| Forshed et al 2008 |  | Net growth including recruitment and mortality of trees |
| Mendez-Toribio et al 2019 |  | Net growth including recruitment and mortality of trees |
| Philipson et al 2020 |  | Net growth including recruitment of trees |
| Lussetti 2017 | Missing mean growth data | - |
| Okali 1987 |  |  |
| Strugnell 1939 |  |  |
| Vidal et al 2016 |  |  |
| West et al 2014 |  |  |
| Do et al 2019 | No control data | Not appropriate controls and sampling to assess impact of liana removal on tree growth |
| Inada and Widiyatno et al 2017 |  | - |
| Pena-Claros and Fredericksen et al 2008 |  |  |
| Pena-Claros and Peters et al 2008 |  |  |
| Schulze, 2003 |  |  |
| Vidal et al 2002 |  |  |
| de Avila, 2016 |  |  |
| Roopsind et al 2008 |  |  |

**Table 4: List of studies included in meta-analysis and summary of study details.**

| **Study ID** | **Author & Year** | **Country** | **Latitude** | **Longitude** | **Elevation (m.a.s.l)** | **Total rainfall (mm year^-1^)** | **Mean temp (°C)** | **Dry season length (months)** | **Disturbance type *^a^*** | **Removal before disturbance (months)** | **Disturbance before removal (months)** | **Removal number (max) *^b^*** | **Removal method *^c^*** |
| --- | --- | --- | --- | --- | --- | --- | --- | --- | --- | --- | --- | --- | --- |
| 2 | Alvarez-Cansino et al 2015 | Panama | 9.11 | -79.85 | 82 | 2866 | 26.3 | 4 | S | NA | 660 | 4 | all |
| 33 | Campanello et al 2007 | Argentina | -25.97 | -54.22 | 255 | 1883 | 21.6 | 0 | SL | NA | 2 | 1 | all |
| 43 | Cesar et al 2016 | Brazil | -22.71 | -47.63 | 555 | 1583 | 22.4 | 6 | SL | NA | 420 | 3 | all |
| 69 | Freitas Xavier et al 2017 | Brazil | -15.85 | -48.96 | 776 | 1732 | 23.7 | 5 | SL | NA | 132 | 0 | all |
| 74 | Gerwing 2001 | Brazil | -3 | -50 | 65 | 2325 | 27.8 | 5 | SL | NA | 96 | 1 | all |
| 79 | Grauel et al 2004 | Panama | 8.12 | -77.87 | 13 | 1495 | 26.8 | 5 | SL | NA | 120 | 0 | all |
| 82 | Grogan et al 2009 | Brazil | -7.83 | -50.27 | 246 | 1878 | 26.5 | 4 | SL | NA | 48 | 1 | tree centred |
| 86 | van der Heijden et al 2015 | Panama | 9.11 | -79.85 | 82 | 2896 | 26.5 | 4 | S | NA | 720 | 13 | all |
| 87 | van der Heijden et al 2019 | Panama | 9.11 | -79.85 | 82 | 2870 | 26.6 | 4 | S | NA | 720 | 21 | all |
| 123 | Lussetti et al 2016 | Malaysia | 4.55 | 117.03 | 533 | 2743 | 24.7 | 0 | SL | 12 | NA | 0 | all |
| 125 | Marshall et al 2017 | Tanzania | -7.82 | 36.98 | 290 | 1144 | 21.5 | 7 | SL+S | NA | 384 | 10 | tree centred |
| 126 | Martinez-Izquierdo et al 2016 | Panama | 9.11 | -79.85 | 82 | 2848 | 26.4 | 4 | S | NA | 720 | 12 | all |
| 133 | Mills et al 2019 | Belize | 17.25 | -89 | 80 | 2048 | 25.7 | 3 | NA | 1 | NA | 0 | tree centred |
| 139 | O'Brien et al 2019 | Malaysia | 5.09 | 117.64 | 144 | 2964 | 25.7 | 0 | SL | NA | 240 | 1 | all |
| 146 | Parren 2003 | Cameroon | 3 | 10 | 475 | 2393 | 25.4 | 4 | SL+S | 9 | NA | 0 | all |
| 177 | Schnitzer et al 2010 | Panama | 9.17 | -79.85 | 86 | 2943 | 26.3 | 4 | S | NA | 600 | 27 | all |

*Table 4 continued:*

| **Study ID** | **Author & Year** | **Country** | **Latitude** | **Longitude** | **Elevation (m.a.s.l)** | **Total rainfall (mm year^-1^)** | **Mean temp (°C)** | **Dry season length (months)** | **Disturbance type(s) *^a^*** | **Removal before disturbance (months)** | **Disturbance before removal (months)** | **Removal repeats (max) *^b^*** | **Removal method *^c^*** |
| --- | --- | --- | --- | --- | --- | --- | --- | --- | --- | --- | --- | --- | --- |
| 198 | de Souza et al 2014 | Brazil | -3.62 | -48.62 | 141 | 2490 | 28 | 4 | SL | NA | 1 | 0 | tree centred |
| 205 | Taffarel et al 2014 | Brazil | -3.62 | -48.62 | 141 | 2490 | 28 | 4 | SL | NA | 1 | 0 | tree centred |
| 219 | Verwer et al 2008 | Bolivia | -15.78 | -62.92 | 233 | 1133 | 24.7 | 6 | SL | NA | 1 | 0 | tree centred |
| 224 | Wright et al 2015 | Panama | 9.17 | -79.85 | 86 | 2920 | 26.3 | 4 | S | NA | 720 | 8 | all |
| 226 | Perez-Salicrup 2001 | Bolivia | -14.75 | -62 | 194 | 1237 | 25.7 | 6 | NA | NA | NA | 0 | all |
| 227 | Perez-Salicrup et al 2000 | Bolivia | -14.75 | -62 | 194 | 1347 | 25.6 | 6 | NA | NA | NA | 0 | tree centred |
| 246 | Estrada-Villegas et al 2020 | Panama | 9.2 | -79.75 | 198 | 2871 | 26.7 | 4 | S | NA | 270 | 4 | all |
| 271 | Venegas-Gonzalez et al 2020 | Brazil | -22.71 | -47.63 | 555 | 1501 | 22.3 | 4 | SL | NA | 384 | 0 | all |
| 323 | Estrada-Villegas et al 2021 | Panama | 7.43 | 80.18 | 73 | 2296 | 27.5 | 2 | SL+S | NA | 420 | 4 | all |

***^a^*** Type of forest disturbance: SL = selectively logged, S = secondary forest (forest regrowth after clear cutting), NA = undisturbed forest

*^b^* Number of times climber removal was repeated

*^c^* Removal of all climbers from the plot/stand (“all”), or removal of climbers from focal trees only (“tree centred”)

**References:**

Livoreil, B., Glanville, J., Haddaway, N. R., Bayliss, H., Bethel, A., De Lachapelle, F. F., Robalino, S., Savilaakso, S., Zhou, W., Petrokofsky, G., & Frampton, G. (2017). Systematic searching for environmental evidence using multiple tools and sources. *Environmental Evidence*, *6*(1), 1–14. https://doi.org/10.1186/s13750-017-0099-6

**Appendix B: Data extraction and explanatory variables**

*Tree growth response data*

We used relative growth rate (RGR) where possible to standardise tree growth by tree size, accounting for differences in growth rate across life stages and sizes. Tree growth was typically reported as change in diameter (*d*) at breast height (dbh) (20 studies), but also as change in biomass, basal area, or height (six studies). Some studies calculated RGR using equation (1) or (2) below, and we calculated RGR using the same equations where possible if RGR was not given. When initial size was not available, but the study used a narrow tree size class (e.g., 5-10 cm dbh), RGR was estimated using the mid-point of the size class. RGR was not available nor could be estimated for seven studies. Whether a study response variable is based on RGR is included in the study quality index (see Table 2).

$$\begin{aligned} RGR=\frac{\ln\left( d_{1} \right)-\ln{(d}_{0})}{t_{1}-t_{0}} \#\left( 1 \right) \end{aligned}$$

$$\begin{aligned} RGR=\left( \frac{1}{d_{0}} \right)\left( \frac{d_{1}- d_{0}}{t_{1}-t_{0}} \right) \#\left( 2 \right) \end{aligned}$$

*d_0_* initial diameter/basal area/biomass/height*; d_1_* final diameter/basal area/biomass/height*;*

*t_0_* initial time*;* t_1_ final time.

If a study reported multiple growth rates for individual species or subsets of species, we aggregated them to obtain a single mean growth rate per study. Some studies measured the growth of the whole tree community in the experimental site, while others only measured a subset of species. We included the number of species measured in each study as a covariate. If a study did not state the number of species measured, we took the average number of species in the ‘whole community’ or ‘subset community’ from the other studies in the meta-analysis (160 and 10 species, respectively).

*Biomass response data*

We quantified the effect of climber removal on biomass from a subset of data that met two criteria: 1) the outcome of climber removal on biomass was available or could be calculated, and 2) data was from trees 5 cm dbh or greater as they have the greatest contribution to aboveground biomass. This resulted in 12 studies. Biomass was already reported in four of these studies, but we calculated biomass in the remaining studies from the tree growth data. In studies for which we had individual tree diameter measurements (N=5), we estimated the biomass using a pan-tropical allometry for moist tropical forests using the equation: $\begin{aligned} AGB={\exp\left( -2.024-0.896E+\log\left( W \right)+2.795\log\left( D \right)-0.0461[log(D \right)]}^{2}) \#\left( 3 \right) \end{aligned}$

(Chave *et al.*, 2014)

$E$ is a variable that represents climatic factors for each region that constrain the height-diameter relationship when height measurements are not available; $W$ is wood density (g cm^3^); $D$is the diameter (cm). *W* was acquired from the global wood density database (Zanne *et al.*, 2009). We used wood density specific to species and region where possible, followed by median wood density for genus and region, or median wood density for the dataset when species name was missing or did not match the wood density database. When individual tree level measurements were unavailable but narrow diameter classes were reported (N=3 studies), we estimated change in biomass using the mid-point of the reported diameter class.

*Missing data*

We contacted authors when there were missing values for growth rate, variance, sample size, or other study design data. If data were still not available but presented in a figure, we extracted values using DigitizeIT software (Bormann, 2020). Studies were excluded from analysis if mean tree growth or biomass increase was still unavailable. However, we used multiple imputation to estimate missing variances following Kambach *et al.*, (2020) and using the *mice* R package (van Buuren and Groothuis-Oudshoorn, 2011). We imputed missing variance using the linear relationship between variance, mean growth and sample size because mean and sample size explained a high percentage of the variance. We ran 10 imputation iterations, generating 10 tree growth and biomass datasets.

*Variables relating to the efficacy of climber removal*

Climate measures were obtained for each study using high-resolution (0.5 x 0.5 degree) data from the Climate Research Unit (CRU) (Harris *et al.*, 2020). The variables calculated were mean annual temperature, total annual precipitation, presence of dry season, dry season length (dry season defined as any month <100mm total rainfall), total dry season precipitation, and mean dry season temperature. We used the International Centre for Tropical Agriculture (CIAT) dataset to obtain elevation for each study site, using the site coordinates with a 1 km buffer (Jarvis *et al.*, 2008). All other potential explanatory variables were extracted directly from the paper.

**Table 1: Criteria for ordinal study quality score.** Study quality is included in models as to account for variation due to study design.

| **Total score and quality category** | **Score*** | **Criteria** |
| --- | --- | --- |
| Low  <6 | 1  1  1 (0.33 per part) | Sample size <4  Sample area <1000 / #trees <50  Design   - just post-treatment + not RGR; - >10km between treatment and control plots; - different forest disturbance history in treatment and control plots |
| Med  6-7 | 2  2  2 (0.66 per part) | Sample size 4-10  Sample area 1000-10,000 / #trees 50-100  Design   - before/after data without RGR (or vice-versa); - 1-10 km between treatment and control plots; - Different disturbance type/ logging type/ secondary forest age between treatment and control plots |
| High  >=8 | 3  3  3 (1 per part) | Sample size >10  Sample area >10,000 / #trees >100  Design   - before/after design + RGR; - <1km between treatment and control; - no differences in disturbance history between treatment and control |

***** score of .99 is rounded up. E.g., 7.99 rounds up to 8

**Table 2: Explanatory parameters that could not be included in the tree growth models for Objective 2 (main nor supplementary models).**

| **Driver of variation** | **Parameter** | **Reason not assessed in Objective 2** |
| --- | --- | --- |
| Region and climate | Region | Too few studies in each category level / correlated with other variable |
|  | Continent | Too few studies in each category level / correlated with other variable |
|  | KPG Climate Classification | Too few studies in each category level / correlated with other variable |
|  | Seasonality | Too few studies in each category level / correlated with other variable |
| Forest type and disturbance | Forest disturbance context | Too few studies in each category level / correlated with other variable |
|  | Secondary forest age | Too few studies in secondary forest |
|  | Liana abundance | Too few studies reporting data |
|  | Tree species or functional group | Too few studies reporting data |
| Liana removal method | LR pre or post disturbance | Too few studies in each category level / correlated with other variable |
|  | Time LR pre disturbance | Too few studies |

**References**

Bormann, I. (2020). *DigitizeIt* (2.5.3).

Chave, J., Réjou-Méchain, M., Búrquez, A., Chidumayo, E., Colgan, M. S., Delitti, W. B. C., Duque, A., Eid, T., Fearnside, P. M., Goodman, R. C., Henry, M., Martínez-Yrízar, A., Mugasha, W. A., Muller-Landau, H. C., Mencuccini, M., Nelson, B. W., Ngomanda, A., Nogueira, E. M., Ortiz-Malavassi, E., … Vieilledent, G. (2014). Improved allometric models to estimate the aboveground biomass of tropical trees. *Global Change Biology*, *20*(10), 3177–3190. <https://doi.org/10.1111/gcb.12629>

Harris, I., Osborn, T. J., Jones, P., & Lister, D. (2020). Version 4 of the CRU TS monthly high-resolution gridded multivariate climate dataset. *Scientific Data*, *7*(1), 1–18. https://doi.org/10.1038/s41597-020-0453-3

Jarvis, A., Guevara, E., Reuter, H. I., & Nelson, A. D. (2008). *Hole-filled SRTM for the globe : version 4 : data grid*. CGIAR Consortium for Spatial Information. http://srtm.csi.cgiar.org/

Kambach, S., Bruelheide, H., Gerstner, K., Gurevitch, J., Beckmann, M., & Seppelt, R. (2020). Consequences of multiple imputation of missing standard deviations and sample sizes in meta-analysis. *Ecology and Evolution*, *10*(20), 11699–11712. https://doi.org/10.1002/ece3.6806

Livoreil, B., Glanville, J., Haddaway, N. R., Bayliss, H., Bethel, A., De Lachapelle, F. F., Robalino, S., Savilaakso, S., Zhou, W., Petrokofsky, G., & Frampton, G. (2017). Systematic searching for environmental evidence using multiple tools and sources. *Environmental Evidence*, *6*(1), 1–14. https://doi.org/10.1186/s13750-017-0099-6

van Buuren, S., & Groothuis-Oudshoorn, K. (2011). mice: Multivariate Imputation by Chained Equations. *R. Journal of Statistical Software*, *45*(3), 1–67.

Zanne, A. E., Lopez-Gonzalez, G., Coomes, D. A., Ilic, J., Jansen, S., Lewis, S. L., Miller, R. B., Swenson, N. G., Wiemann, M. C., & Chave, J. (2009). Data from: Towards a worldwide wood economics spectrum. Dataset, Dryad. https://doi.org/https://doi.org/10.5061/dryad.234

**Appendix C: Model specifications**

**Table 1: Model specification for quantifying the magnitude of climber removal efficacy (objective 1) and for assessing drivers of variation in efficacy (objective 2)**. All models used SMD (standardised mean difference) effect size as response variable, were weighted by 1/SMD variance, and included study as random effect. ‘Nuisance’ variables of study quality, number of species used to calculate mean growth, and time elapsed between removal and measurement were also included as fixed effects in all models.

| ***Objective number*** | ***Objective*** | ***Response variable*** | ***Explanatory variable*** |
| --- | --- | --- | --- |
| **Objective 1.1** | **Quantify magnitude of, and variance in, efficacy of CR to promote tree growth** | - Tree growth | - *None* |
| **Objective 1.2** | **Quantify magnitude of, and variance in, efficacy of CR to promote aboveground biomass accumulation** | - AGB change | - *None* |
| **Objective 2.1** | **Assess the drivers of variation in efficacy of CR to promote tree growth** | - Tree growth | - Logged forest (Y/N), - Repeat removal (Y/N), - Elevation, - Dry season length, - Annual precipitation, - Average temperature - Removal method (remove climbers on focal tree / climbers from entire area) |
| **Objective 2.2a** | **Assess the drivers of variation in efficacy of CR to promote aboveground biomass accumulation** | - AGB change | - Repeat removal (number of times repeated) |
| **Objective 2.2b** |  |  | - Repeat removal (Y/N) |
| **Objective 2.2c** |  |  | - Repeat removal (number of times repeated) - Time since disturbance (time between disturbance and treatment) |

**Table 2: Supplementary models assessing additional drivers of variation in climber removal efficacy for tree growth (objective 2.1) which could not be included in the main model**. Each model includes an additional explanatory variable that could not be assessed in the objective 2 model in the main text, highlighted in bold. All models used SMD (standardised mean difference) as response variable, were weighted by 1/SMD variance and included study as random effect. ‘Nuisance’ variables study quality, number of species used to calculate mean growth and time elapsed between removal and measurement were also included as fixed effects in all models.

| ***Additional variable assessed*** | ***Explanatory variable*** |
| --- | --- |
| **Latitude** | - Logged forest (Y/N), - Repeat removal (Y/N), - Elevation, - Dry season length, - Annual precipitation, - Average temperature - **Latitude** |
| **Number of times removal repeated** | - Logged forest (Y/N), - Elevation, - Dry season length, - Annual precipitation, - Average temperature, - **Repeat removal (number of times repeated)** |
| **Time since disturbance**  **(post-treatment studies only)** | - Logged forest (Y/N), - Repeat removal (Y/N), - Elevation, - Average temperature, - **Time since disturbance (time between disturbance and treatment)** |
| **Dry season climate**  **(dry season studies only)** | - Logged forest (Y/N), - Repeat removal (Y/N), - Elevation, - Dry season length, - **Dry season precipitation,** - **Dry season temperature** |

**Appendix D: Additional analyses**

*Sensitivity analysis and publication bias*

We found some evidence for publication bias in our meta-analysis. While there was no relationship between publication year and effect size for tree growth and biomass analyses (Appendix D, Figure 1, 2), the funnel plots of effect size against variance were asymmetric (Appendix D, Figure 3, 4), and the Eggers test indicates a significant relationship between effect size and variance (p < 0.01 and p < 0.0001 for tree growth and biomass, respectively). However, fail-safe numbers indicate that the meta-analysis results are robust. According to the Rosenberg and Rosenthal methods, there would need to be between 310-560 additional studies with null results to reduce the significance level of the tree growth summary effect size to above alpha = 0.05, and 118-294 for the biomass effect size. Alternatively, according to the Orwin method, there would need to be 26 and 12 further studies with null results to reduce the tree growth and biomass summary effect sizes by half, respectively.


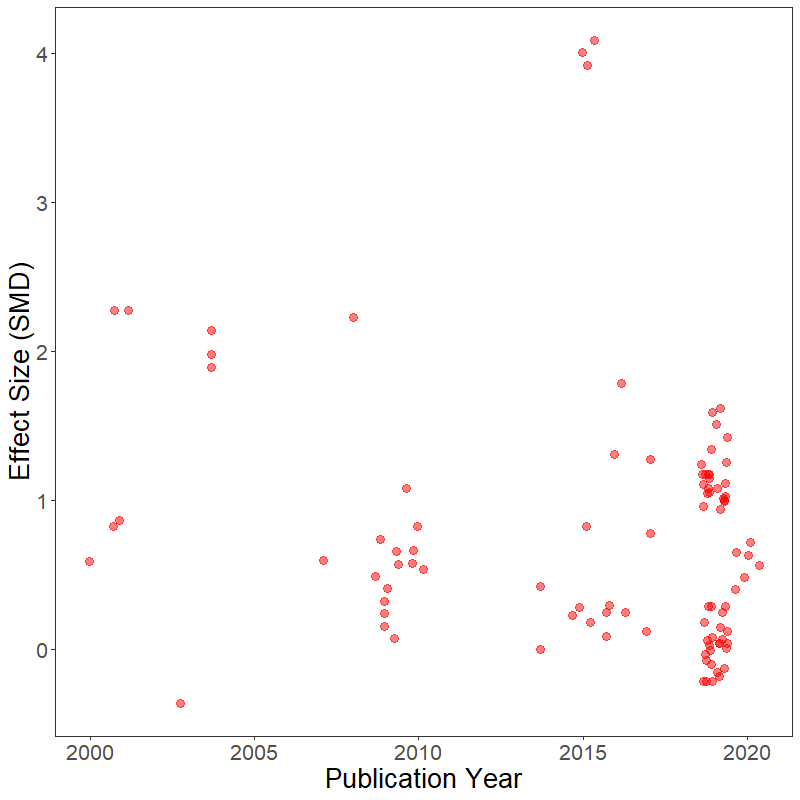


**Figure 1: Relationship between publication year and tree growth effect size.** Publication year is plotted against average study tree growth effect size (average of individual effect sizes in each study), predicted from growth summary ES model (objective 1.1), to assess publication bias.


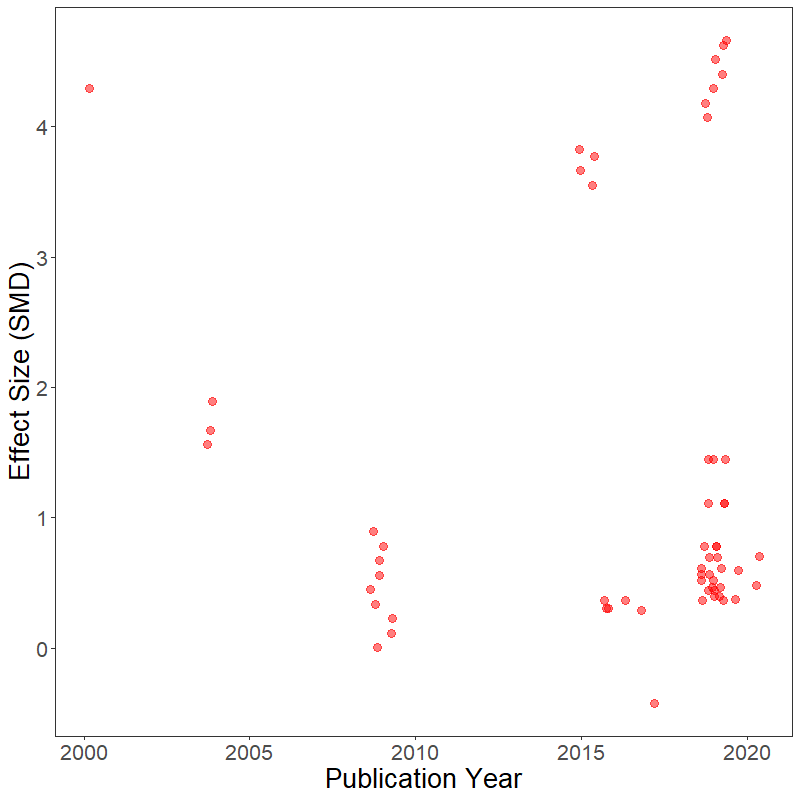


**Figure 2**: **Relationship between publication year and AGB effect size.** Publication year is plotted against average study AGB effect size (average of individual effect sizes in each study), predicted from growth summary ES model (objective 1.2), to assess publication bias.


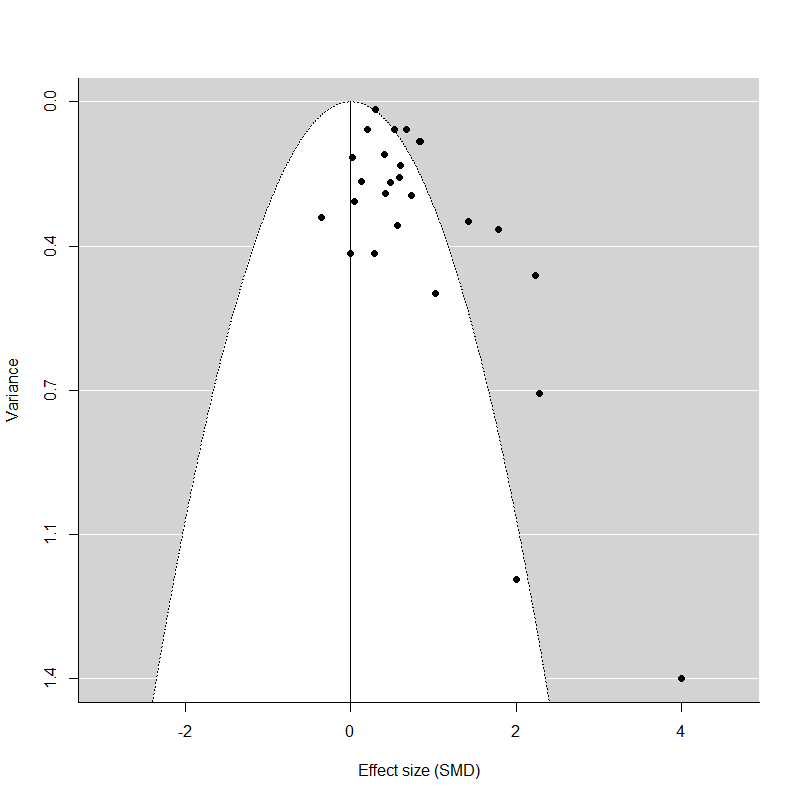


**Figure 3:** Funnel plot of study tree growth effect size (SMD) (average of individual effect sizes in each study predicted from objective 1.1 model) against effect size variance.


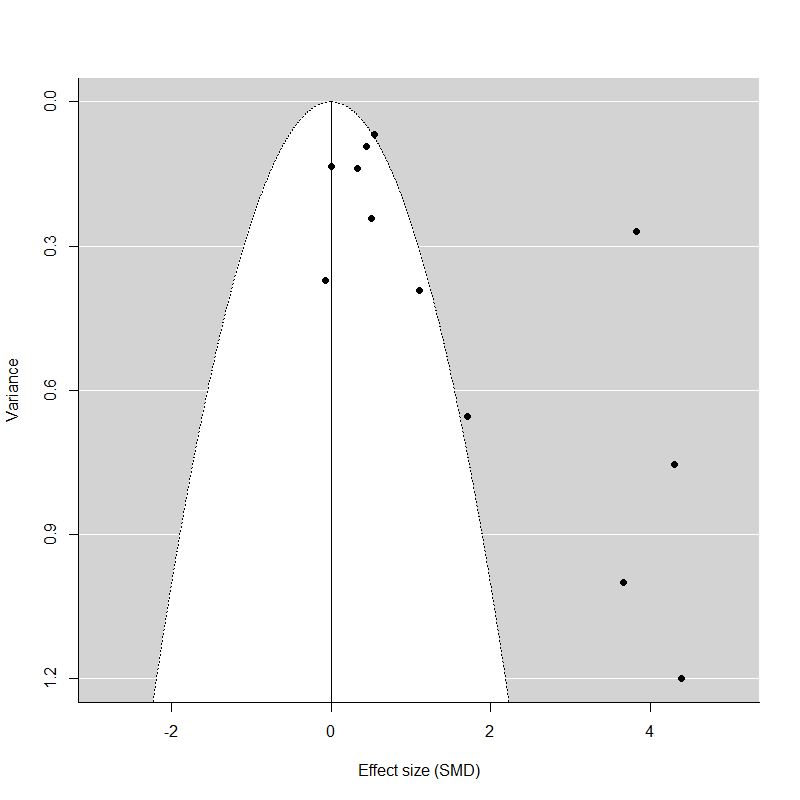


**Figure 4**: Funnel plot of biomass study effect size (SMD) (average of individual effect sizes in each study predicted from objective 1.2 model) against effect size variance.


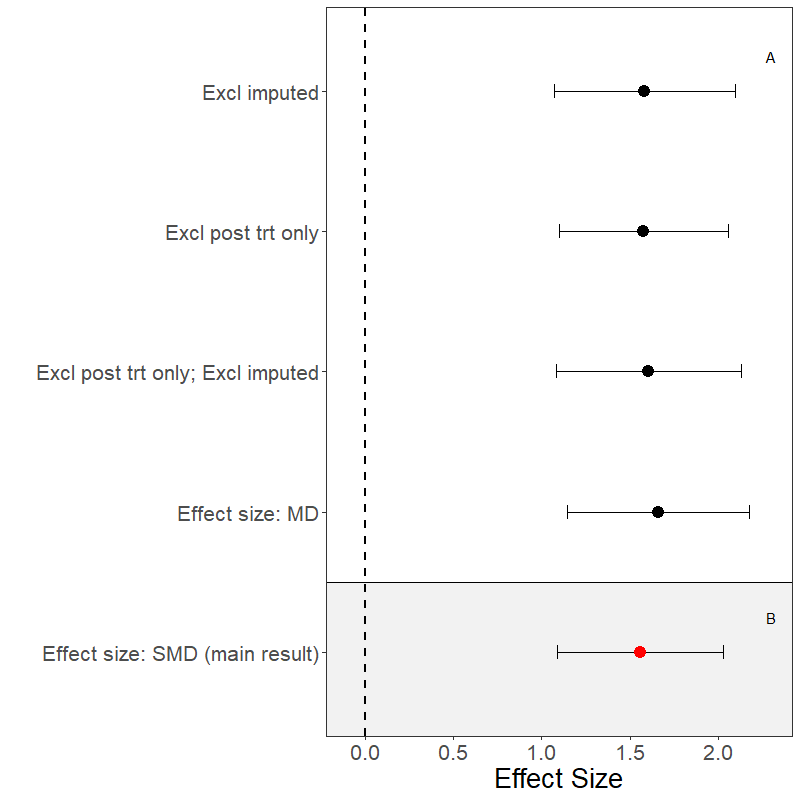


**Figure 5:** Efficacy of climber removal for enhancing tree growth depending on data type and effect size, calculated using model for objective 1.1 (response variable tree growth) with different combinations of data: a) excluding imputed data, excluding studies with just post-treatment data, excluding both data, using MD as effect size rather than SMD, b) final tree growth summary effect size used in the manuscript: including imputed data calculated using linear regression, studies with just post-treatment data and using SMD.

**Table 1: Magnitude and direction of climber removal efficacy on tree growth without van der Heijden *et al* (2015) outlier.** Results of models for Objective 1.1 (tree growth) without outlier. ‘Tree growth Effect Size (ES)’ are the intercept of the model and show the number of times greater tree growth with climber removal versus untreated control plots. Results are the average of 10 Linear Mixed Models using 10 datasets imputed using linear regression, including the study with just post-treatment data (N=25 studies). See Appendix C for full description of models. Bolded effect sizes indicate level of significance at either 0.05,0.01, or 0.001.

| ***Objective*** | ***Fixed effect*** | ***Estimate (SE)*** | ***Confidence Intervals*** | ***Degrees of Freedom*** |
| --- | --- | --- | --- | --- |
| *Objective 1.1: Tree growth* | ***Tree growth ES*** | **1.38 (0.19)***** | 0.98 – 1.78 | 27 |
|  | ***Study quality High:Low*** | **-1.12 (0.34)**** | -1.81 – -0.43 | 64 |
|  | ***Study quality High:Med*** | **-1.11 (0.14)***** | -1.39 – -0.83 | 86 |
|  | *Number of species* | 0.00 (0.00) | 0.00 – 0.00 | 89 |
|  | ***Time elapsed since removal*** | **0.01 (0.00)***** | 0.00 – 0.01 | 89 |

**p<0.05, **p<0.01. ***p<0.001*

*
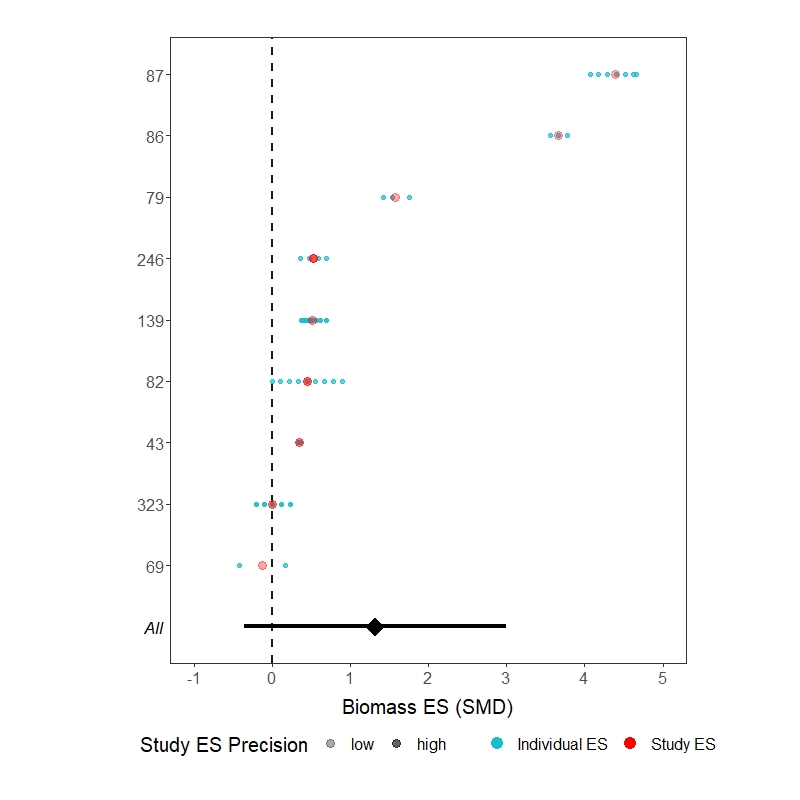
*

**Figure 6:** Overall, individual, and study average effect sizes (ES) of climber removal for promoting AGB accumulation, without including imputed data. Blue dots are individual effect sizes within a study, predicted from the models for Objective 1.2 and averaged for all imputed datasets. Red circles are the study ES (the average of the individual ES); the size of the circle represents precision of the study ES and is proportional to the inverse of the variance of the individual effect sizes, averaged by study. The black diamond at the bottom of each figure is the overall summary effect size of climber removal for promoting tree growth and biomass, taken from the intercept of the models for Objective 1.2 when continuous covariates are at their mean value and study quality is set to high; error bar shows 95% credible intervals.

**Table 2:** **Additional** **drivers of variation in the efficacy of climber removal for tree growth.** Table shows results of supplementary models for objective 2.1, averaged from 10 Linear Mixed Models using 10 imputed datasets (imputed using linear regression) and including one study with just post-treatment data (N=26 studies). Response variable is tree growth, see full model details in Appendix C.

| *Model* | *Explanatory parameter* | *Estimate (SE)* | *Degrees of Freedom* |
| --- | --- | --- | --- |
| a | ***Time elapsed since removal*** | **0.28 (0.07)***** | 85 |
|  | *Repeat removal (Y/N)* | -0.34 (0.20) | 91 |
|  | *Logged forest* | -0.30 (0.61) | 16 |
|  | *Dry season length* | 0.56 (0.38) | 16 |
|  | *Annual precip* | 0.26 (0.40) | 17 |
|  | *Annual temp* | -0.7 (0.27) | 18 |
|  | *Elevation* | -0.26 (0.29) | 22 |
|  | *Latitude* | -0.03 (0.34) | 19 |
| b | ***Time elapsed since removal*** | **0.21 (0.07)**** | 84 |
|  | *Repeat removal (number)* | 0.12 (0.13) | 87 |
|  | *Logged forest* | -0.32 (0.60) | 17 |
|  | *Dry season length* | 0.39 (0.40) | 20 |
|  | *Annual temp* | 0.15 (0.27) | 21 |
|  | *Annual precip* | 0.02 (0.37) | 20 |
|  | *Elevation* | -0.04 (0.28) | 22 |
| c | ***Time elapsed since removal*** | **0.31 (0.09)**** | 60 |
|  | *Repeat removal (Y/N)* | -0.37 (0.23) | 68 |
|  | *Time since disturbance* | -0.01 (0.22) | 15 |
|  | *Dry season length* | 0.53 (0.36) | 13 |
|  | *Elevation* | -0.29 (0.32) | 14 |
|  | *Annual temp* | -0.01 (0.24) | 15 |
| d | ***Time elapsed since removal*** | **0.17 (0.07)*** | 43 |
|  | *Repeat removal (Y/N)* | 0.04 (0.22) | 53 |
|  | *Logged forest* | 0.03 (0.54) | 14 |
|  | *Dry season length* | 0.95 (0.58) | 14 |
|  | *Elevation* | -0.29 (0.31) | 15 |
|  | *Dry season annual temp* | 0.02 (0.34) | 15 |
|  | *Dry season annual precip* | -0.15 (0.29) | 14 |

**p<0.05, **p<0.01. ***p<0.001*
